# A novel progesterone receptor membrane component (PGRMC) in the human and swine parasite *Taenia solium*: implications to the host-parasite relationship

**DOI:** 10.1186/s13071-018-2703-1

**Published:** 2018-03-09

**Authors:** Hugo Aguilar-Díaz, Karen E. Nava-Castro, Galileo Escobedo, Lenin Domínguez-Ramírez, Martín García-Varela, Víctor H. del Río-Araiza, Margarita I. Palacios-Arreola, Jorge Morales-Montor

**Affiliations:** 1 Centro Nacional de Investigación Disciplinaria en Parasitología Veterinaria, Instituto Nacional de Investigaciones Forestales Agrícolas y Pecuarias INIFAP, CP 62550 Jiutepec, Morelos Mexico; 2 Laboratorio de Genotoxicología y Medicina Ambientales. Departamento de.Ciencias Ambientales. Centro de Ciencias de la Atmósfera, Universidad Nacional Autónoma de México, 04510 Ciudad de Mexico, Mexico; 3 Unidad de Medicina Experimental, Hospital General de México “Dr. Eduardo Liceaga”, 06726 México DF, Mexico; 4 Departamento de Ciencias Químico-Biológicas, Universidad de las Américas Puebla, Sta. Catarina Mártir, Cholula, C.P 72810 Puebla, Mexico; 5 Instituto de Biología, Universidad Nacional Autónoma de México, CP 04510 Ciudad de Mexico, DF Mexico; 6 Departamento de Inmunología, Instituto de Investigaciones Biomédicas, Universidad Nacional Autónoma de México, AP 70228, 04510 Ciudad de Mexico, DF Mexico

**Keywords:** *Taenia solium*, Cysticerci, Parasite, Helminth, PGRMC, Hormone receptors, Progesterone

## Abstract

**Background:**

We have previously reported that progesterone (P_4_) has a direct in vitro effect on the scolex evagination and growth of *Taenia solium* cysticerci. Here, we explored the hypothesis that the P_4_ direct effect on *T. solium* might be mediated by a novel steroid-binding parasite protein.

**Methods:**

By way of using immunofluorescent confocal microscopy, flow cytometry analysis, double-dimension electrophoresis analysis, and sequencing the corresponding protein spot, we detected a novel PGRMC in *T. solium*. Molecular modeling studies accompanied by computer docking using the sequenced protein, together with phylogenetic analysis and sequence alignment clearly demonstrated that *T. solium* PGRMC is from parasite origin.

**Results:**

Our results show that P_4_ in vitro increases parasite evagination and scolex size. Using immunofluorescent confocal microscopy, we detected that parasite cells showed expression of a P_4_-binding like protein exclusively located at the cysticercus subtegumental tissue. Presence of the P_4_-binding protein in cyst cells was also confirmed by flow cytometry. Double-dimension electrophoresis analysis, followed by sequencing the corresponding protein spot, revealed a protein that was previously reported in the *T. solium* genome belonging to a membrane-associated progesterone receptor component (PGRMC). Molecular modeling studies accompanied by computer docking using the sequenced protein showed that PGRMC is potentially able to bind steroid hormones such as progesterone, estradiol, testosterone and dihydrodrotestosterone with different affinities. Phylogenetic analysis and sequence alignment clearly demonstrated that *T. solium* PGRMC is related to a steroid-binding protein of *Echinoccocus granulosus*, both of them being nested within a cluster including similar proteins present in platyhelminths such as *Schistocephalus solidus* and *Schistosoma haematobium*.

**Conclusion:**

Progesterone may directly act upon *T. solium* cysticerci probably by binding to PGRMC. This research has implications in the field of host-parasite co-evolution as well as the sex-associated susceptibility to this infection. In a more practical matter, present results may contribute to the molecular design of new drugs with anti-parasite actions.

**Electronic supplementary material:**

The online version of this article (10.1186/s13071-018-2703-1) contains supplementary material, which is available to authorized users.

## Background

Human neurocysticercosis and porcine cysticercosis are caused by the metacestode stage of the cestode parasite *Taenia solium*. Neurocysticercosis is still a serious human health problem, whereas porcine cysticercosisis is a veterinary problem in many developing countries and mainly in underdeveloped countries. Neurocysticercosis approximately affects 50 million people worldwide [1–4]. Human neurocysticercosis is considered as an emergent disease in the USA [4]. Swine cysticercosis leads to major economic losses and also promotes continuity of the infectious cycle in humans [5, 6].

One of the most important aspects during *T. solium* infection is the evagination process of *T. solium* cysticerci that takes place in the human gut. Evagination is the key step that will release the adult worm, which can produce thousands of eggs. Infective eggs released with human stools can contaminate the environment and infect pigs, where the eggs rapidly differentiate into cysticerci. Cysticerci are mainly located in the skeletal muscles in pigs or brain tissue in humans, where the most severe symptoms are observed in patients with neurocysticercosis [3, 7].

Some reports show that sex hormones are involved in favoring cysticercosis in female pigs, revealing that the frequency of *T. solium* cysticercosis in pigs is increased during pregnancy, which is characterized by a significant elevation in progesterone levels [8, 9]. As a consequence, the sex hormones might affect the course of *T. solium* parasite infection [10–14]. Furthermore, we have previously found that in vitro treatment with progesterone (P_4_ hereinafter) increases evagination and growth of *T. solium* cysticerci [15], thus demonstrating a direct effect of P_4_. P_4_ effects could be mediated by the presence of a putative progesterone-binding protein in the parasite resembling either a nuclear classical progesterone receptor (PR), or a membrane receptor. In *T. crassiceps*, another related cestode, we have also demonstrated that P_4_ treatment increases parasite loads 2-fold in females and 3-fold in males, as compared to controls [16]. P_4_ is also able to stimulate in vitro reproduction of *T. crassiceps* cysticerci, suggesting the existence of a progesterone-binding protein in *Taenia* spp. [16]. Likewise, P_4_ treatment increases cytoskeleton protein expression including actin, tubulin and myosin in *T. crassiceps*, all prominent components of flame cells belonging to the parasite excretory system [17].

Progesterone can exert its actions through non-genomic mechanisms mediated by the interaction with progesterone-binding membrane proteins including progesterone membrane receptors (mPRs) and the progesterone membrane components (PGRMC-1 and PGRMC-2) [18]. These progesterone membrane components were described to be putative progesterone receptors HPR6.6 (PGRMC-1) and Dg6 (PGRMC-2) in humans [19].

PGRMC-2 is expressed in several tissues, particularly in the placenta and other tissues belonging to the reproductive system. However, it has been shown that PGRMC-2 can be also expressed in non-reproductive tissues such as liver and nervous tissue. Interestingly, PGRMC-2 has been found in other organisms. To this regard, *Caenorhabditis elegans* has been demonstrated to express Vem-1, an analogous protein to PGRMC-2 in mammals [20]. In the specific case of helminth parasites, there are a few studies reporting the presence of PGRMC receptors. In this regard, numerous sex hormone-receptor-related proteins, including PGRMC, small androgen receptor-interacting proteins, progesterone-receptor associated p48 protein and progestin-induced protein, have been found in *S. japonicum* EST data [21, 22]. Likewise, several nuclear receptors and thyroid-hormone-associated proteins were identified in *S. mansoni* EST data [21–23].

The study of this type of progesterone binding molecules might help to expand knowledge on *Taenia* spp. biology in terms of differentiation, reproduction and development, as well as generating possible pharmacological targets that could be used in anti-helminth drug therapy.

The aim of the present study was to explore the hypothesis that direct effects of P_4_ on *T. solium* cysticerci are mediated through a novel steroid-binding parasite protein resembling to PGRMC, by means of in vitro cell cultures, immunofluorescence, flow cytometry, two-dimension electrophoresis (2D-E), protein sequencing, molecular modeling, docking analysis and phylogenetical computational analysis.

## Methods

### Parasites

*Taenia solium* cysticerci were dissected from skeletal muscle of naturally infected pigs. The fibrous capsule that surrounds each parasite was carefully separated using a dissection microscope. Once dissected, cysticerci were placed in tubes containing sterile PBS (1×) supplemented with 100 U/ml antibiotics-fungizone (Gibco, St Louis Missouri, USA). Samples were centrifuged at 800× *g* at 4 °C for 10 min and supernatants were discarded. Pellets containing cysticerci were incubated in Dulbecco’s Modified Medium (DMEM) without fetal calf serum supplementation (Gibco). Collected parasites were centrifuged three times at 800× *g* 10 min and washed with DMEM after each centrifugation. After the final wash, viable parasites (complete and translucent cystic structures) were counted using a binocular microscope. Ten viable cysticerci were then collected and dispensed into single 6-well culture plates (Falcon, BD Labware,) using 5 ml DMEM medium (Gibco) each and then incubated at 37 °C in 5% CO_2_.

### In vitro treatment effects of P_4_ on *T. solium* cysticerci

Evaluation of P_4_ effects on *T. solium* cysticerci was conducted as previously reported by Escobedo et al. [15]. In brief, for in vitro tests, water-soluble P_4_ was obtained from Sigma (St Louis, Missouri) and dissolved in DMEM serum-free culture medium at a final stock concentration of 1 mg/ml and sterilized by filtration using a 0.2 mm Millipore filter. Parasites were cultured in the presence of pure cultured medium or culture medium supplemented with 0.3% ethanol as vehicle (control groups). Also, parasites were separately cultured with 40 nM of P_4_. Cysticerci from all treatments were cultured for 5 days in 5 ml DMEM-medium and incubated at 37 °C in 5% CO_2_. We carried out daily inspections concerning the scolex evagination and worm length. P_4_ reagent was prepared at 100 μl final volume and added to each well containing parasites. The culture media was replaced every 24 h for 5 days. Finally, scolex evagination and worm length were registered using an inverted microscope (Olympus, MO21, Tokyo, Japan). Worm length was considered as the millimetric sum of scolex, neck and strobila.

### Progesterone-binding protein location in *T. solium* cysticerci by immunofluorescence

Cultured *T. solium* cysticerci were washed with PBS 1× and embedded in Tissue Tek (Triangle Biomedical Science, Arizona, USA), and immediately frozen at -80 °C until use. Parasitic tissue sections (5 μm) were fixed with frozen acetone for 10 min, washed 3 times in PBS-Tween 0.3% and blocked for 1 h with PBS containing 1% bovine albumin. Cross-sections were then incubated with a 1:100 dilution of PGRMC polyclonal antibody obtained from *T. spiralis* PGRMC (cloned, sequenced, synthesized and produced by Dr. Romel Hernández-Bello, who kindly donated it to us) for 45 min at 37 °C, washed with PBS and then incubated with Alexa 488-conjugated rabbit anti-mouse antibody (Invitrogen, California, USA) at 1:300 dilution. Control experiments were assessed incubating the 5 μm thick tissue sections in the presence of Alexa 488-conjugated rabbit anti-mouse antibody alone at the same dilution. To eliminate background fluorescence, samples were contrasted with 0.025% Evans Blue for 10 min. After two single washings, samples were mounted in Vectashield mounting medium (Vector Laboratories Inc.,Boston, USA) and examined with a Carl Zeiss epifluorescence microscope (Carl Zeiss, Berlin, Germany).

### Detection of the progesterone-binding protein in tegumental cells of *T. solium* cysticerci by flow cytometry

*Taenia solium* cells were extracted by cysticerci tissue disruption with nylon mesh and syringe plunger from cultured treated and un-treated parasites as previously described [24]. In brief, we first disrupted the entire cisticerci by passing them though a 3 ml syringe and then, the remaining “envelope” was macerated by using a nylon mesh “sandwich” (150 mm) and a syringe plunger. At each step we looked for cells at the microscope and found that this procedure was sufficient for having isolated parasite derived cells [24]. Cells were centrifuged at 1680×*g* for 10 min, and they were resuspended in 100 μl FACS buffer (phosphate-buffered saline (PBS) pH 7.4, 2% SFB, 0.02% NaN_2_) and 100 μl paraformaldehyde 4% in PBS 1× (PF4) was added to fix the cells for 10 min at 37 °C. One millilitre of ice-cold MetOH was added immediately after and incubated for 30 min at 4 °C. Cells were centrifuged at 2240× *g* for 10 min, decanted and washed 3 times with FACS buffer. 30 μl of goat anti-mouse PGRMC polyclonal antibody (donated by Dr. Romel Hernández-Bello) was added and incubated for 10 min. Cells were washed with 1 ml FACS buffer and centrifuged as described above. Finally, cells were incubated with 30 μl of secondary antibody for 10 min. Cells were washed twice, resuspended in 200 μl FACS buffer and stored in dark conditions at 4 °C. Stained cells were registered using a FACSCalibur flow cytometer (Beckton Dickinson, California, USA), and data were analyzed with FlowJo® software.

### PGRMC-like protein sequencing by 2-DE and mass spectrometry

Total protein from cultured *T. solium* cysticerci was extracted and quantified as described before. Protein samples were placed in a buffer containing 8 M urea, 2% CHAPS, 50 mM DTT, IPG pH 3–10 (Bio-Rad, California, USA) and bromophenol blue. Immediately after, protein samples were incubated overnight with the first-dimension gel (Amersham, Amsterdam, Netherlands). Once they were accurately hydrated, the first-dimension gel was isoelectrofocused with a constant voltage on a lineal electric gradient. After this, gel was equilibrated in a buffer containing 6 M urea, 2% SDS, 375 mM Tris pH 8.8, 2% DTT and 20% glycerol for 15 min. Next, the same equilibration process was performed using iodoacetamide 25 mg/ml instead of DTT. Once equilibrated, the gel was separated according to the molecular size of each protein in a second-dimension gel (PAGE-SDS al 12.5%). A single protein spot corresponding to the expected molecular weight and predicted isoelectrical point was cut-out and sequenced by MALDITOF-TOF mass-spectrometry.

### Modeling, docking and molecular dynamics of the putative progesterone receptor membrane component PGRMC

Initial model generation was accomplished by using the hydrophilic segment of PGRMC sequence and submitting it to Rosetta Homology modeling [25]. The sequence used was: SARGKHSNHEKLPNMRKRDFTIQELSQFNGNGPDGRILIAVNGNVFDVTNNGKEFYGKDG PYAIFAGRDASRSLTMFTTDIPPCIEEYDDLSDLTSDQMKSLKEWELQFRERYPLVGKLLSPSEPHHLYESADNEESSQIDALTGSAKPKTDXSNAIYDILCTHCSRPLSILSLFLDFPIVVESSSCAYFVSVCAPVYTSLIVLXVFCFNNAPXFID.

Resulting models clustered close together (RMSD 1.14 to 1.46); only the model ranked first exhibited a surface pocket that could permit a hydrophobic ligand to bind to PGRMC. Thus, after a round of energy minimization, we selected that model to perform ligand docking.

Blind docking was performed using Vina 1.1.2 [26] on a single node from the LNS (Laboratorio Nacional del Supercomputo del Sureste de México). All ligands were obtained from the ZINC database [27] and converted to PDBQT format using the GUI provided by Autodock Tools [28]. Ligands were checked manually against the known chemical structure and all their rotatable bonds were allowed to remain free from restrain during docking. The receptor, i.e. PGRMC, was kept rigid. Docking employed a grid of dimensions 40 × 40 × 40 with a 1 Å grid size. Exhaustiveness was always set to 5000. Analysis of the docking results was performed in PyMOL as well as Daniel Seelinger’s Autodock/Vina plugin.

Molecular Dynamics were performed using AMBER14 [29] in a GPU powered supercomputer. The force field employed was amberff14SB, an all-atom force field, with SPC/E water molecules. The system was solvated in an octahedron and neutralized with sodium ions. Ligands charges were fitted using Gaussian03 and the rest of its parameters taken from GAFF [30]. Simulations were carried out at 300 K; each system was prepared by energy minimizing the water box, then the protein with the water box. The temperature was slowly raised from 0 °C to 300 °C and then a 1 atm pressure applied on the system. The production runs of 100 ns were started after these steps from the docking results. To allow for the equilibration of the ligand in its binding site, only the last 50 ns were employed in further calculations. MMGBSA (Molecular Mechanics/Generalized Born Solvent Accesible [31]) calculations were performed within AMBER. Visualization was done using VMD [32] and figures were prepared with UCSF Chimera [33].

### Alignment and phylogenetic analysis

The sequence obtained in the present work for progesterone receptor component of *T. solium* was aligned separately with other 21 sequences downloaded from the GenBank database, including 4 sequences for Platyhelminthes, 2 for nematodes, 3 for arthropods and 12 for vertebrates including mammals, birds, reptiles and fish. The alignment included 133 characters (amino acids) and was constructed using Clustal W [34] with default parameters and adjusted manually within Mesquite [35]. The genetic divergence among taxa was estimated using uncorrected “p-distance” with MEGA version 6 [36]. The tree was constructed with neighbor-joining method and the nodes of the tree were supported with 10,000 bootstrap replicates.

### Videomicroscopy of live evaginated *T. solium* cyscticerci

Live cysticerci were maintained for five days in DMEM medium supplemented with 25 mM HEPES buffer adjusted to pH 7.2 and 30 mM carbonate salts. Parasites were maintained in a humidified incubator at 37 °C in a 5% CO_2_ environment. Filming motion of live parasites was performed in a microscope (Olympus, MO21).

### Experimental design and statistical analysis

The response variable used in statistical analyses was the number of evaginated scolices that showed growth and motility in all wells treated with P_4_, compared to untreated. Data of the four replicates of each treatment were expressed as the mean ± standard deviation (SD). Data were analyzed using one-way analysis of variance (ANOVA) followed by a *post-hoc* Tukey’s test. Differences were considered significant when *P* < 0.05.

## Results

### In vitro effects of P_4_ on *T. solium* cysticerci

**Fig. 1:**
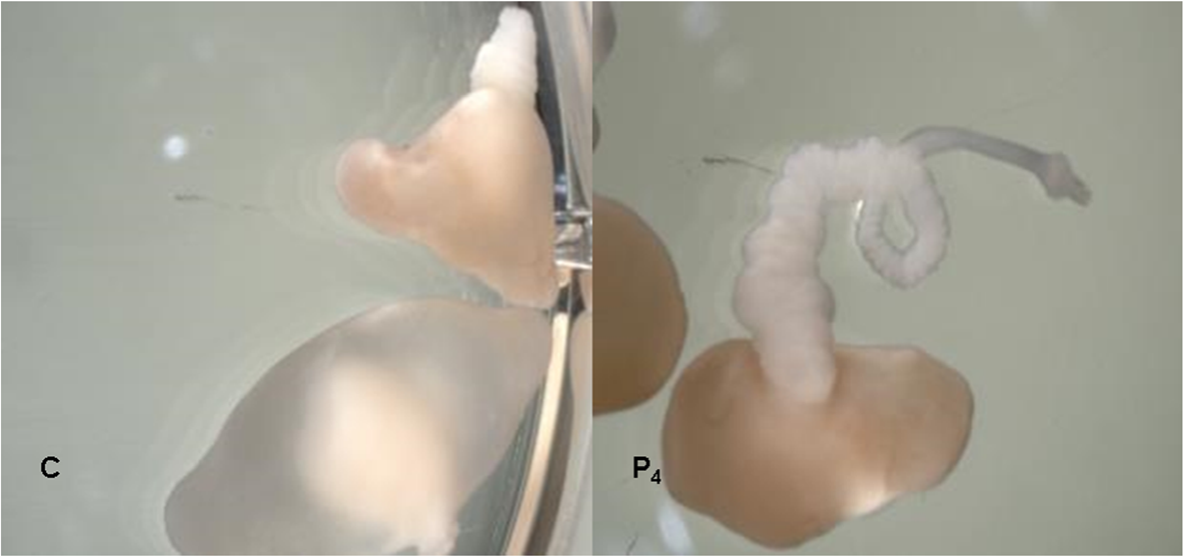
Photographs of *Taenia solium* cysticerci in culture. Control cysticerci evaginated after 5 days in culture (*C*) and cysticerci evaginated after 5 days in culture in the presence of 40 ng of progesterone (P_4_)


As previously reported, we confirm previous results that, when *T. solium* cysticerci were in vitro exposed to P_4_, an increase in the scolex evagination was observed in all treated parasites as compared to control groups, where only 40% of them spontaneously evaginated (Fig. 1). Evaluation of viability in evaginated cysticerci was carried out daily to determinate worm motility in culture conditions. Clearly, progesterone-treated parasites look much better than control ones (Additional file 1: Movie 1). A close up of a *T. solium* cysticerci treated with progesterone, showed a complete differentiation of the scolex, with rostelum, ventosas, and proglottids. The *T. solium* worm is alive and moving, looking for anchorage to the host. Motility of evaginated cysticerci was constant through all days of in vitro culture, as previously reported (Additional file 2: Movie 2).

**Additional file 1: Movie 1.** *T. solium* cysticerci control (right cyst) and treated with progesterone (left cyst). Progesterone treated one is evaginated and larger, and it is translucid and completely motile, looking for an anchor to a tissue. The control one, though evaginated, is smaller and it is opaque. (mov 10000 kb)



**Additional file 2: Movie 2.** Close up of a *T. solium* cysticerci treated with progesterone, in which the complete differentiation of the scolex, showing rostelum, ventosas, and proglottids is shown. The *T. solium* worm is alive and moving, looking for anchorage to the host. (mov 10300 kb)


### Detection of the putative progesterone binding protein in *T. solium* cysticerci by flow cytometry

**Fig. 2:**
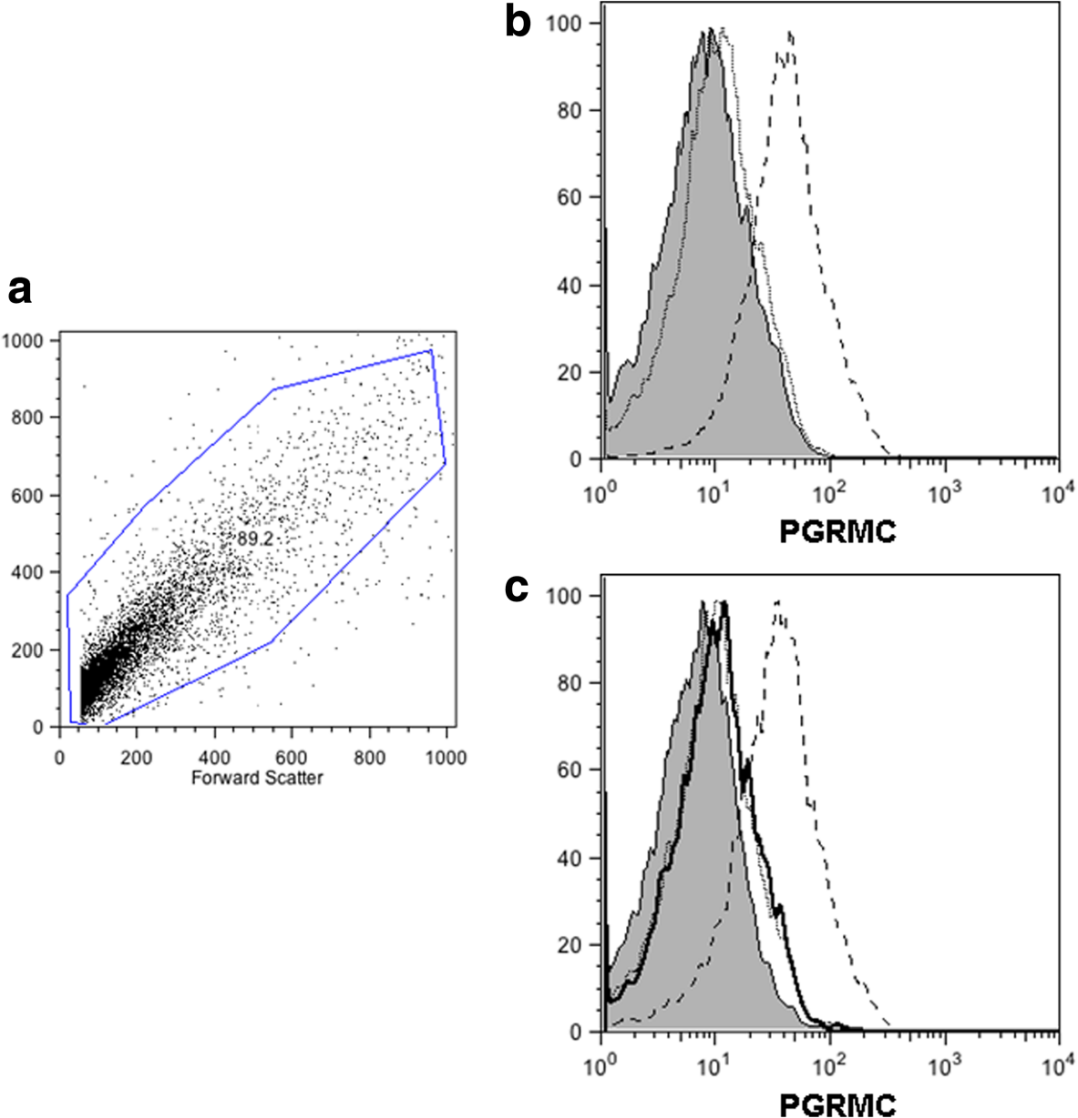
Specific detection of the progesterone-binding protein in *Taenia solium* cysticerci by flow cytometry. FACS analysis in *T. solium* cysticercus cells showed the PGRMC expression. In untreated cysticerci used as controls, very few cells presented a low immuno-fluorescent signal, whereas P_4_-treated cysticerci showed few cells with high immunofluorescent signal related to the receptor expressed in the parasite. **a** Size and complexity of *T. solium* cells. **b** PGRMC expression on *T. solium* cells. **c** PGRMC expression on *T. solium* cells after P_4_ stimulation. Solid lines show un-stained cells in all cases. Dotted lines correspond to unspecific staining of the secondary antibody and long-dashed lines correspond to the specific staining of PGRMC

In Fig. 2a, a dot plot showing the *T. solium* cell size and complexity is shown. In fact, parasite cells were approximately 3-fold smaller and exhibited less complexity than other cell types previously analyzed (Fig. 2a). We found *T. solium* cells expressing the PGRMC in basal conditions (Fig. 2b). Interestingly, the expression pattern of the PGRMC was not altered when parasites were stimulated with progesterone (Fig. 2c).

### Immunolocalization of a putative PR-binding protein in *T. solium* cysticerci

**Fig. 3:**
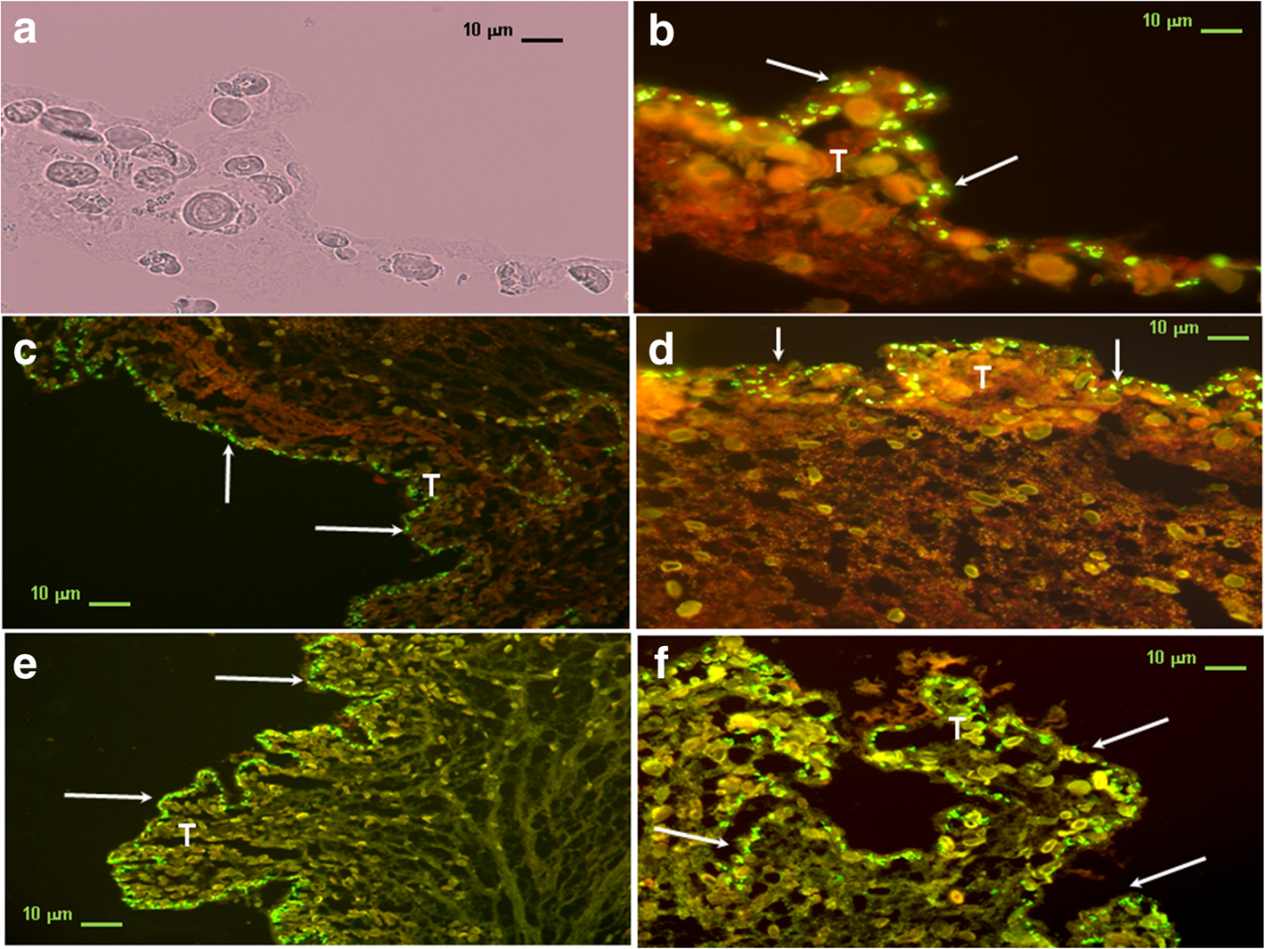
Progesterone-binding membrane protein location in *Taenia solium* cysticerci by immunofluorescence. **a** Representative transversal sections of *T. solium* cysticerci where tegument, sub-tegument and cells are observed with optical microscopy illumination (Nomarski microscopy). **b** Negative control of immunofluorescence using the secondary antibody. **c** Specific detection of progesterone binding membrane protein in parasite (arrows) cells mainly located all along subtegumental tissue (magnification of 100×). **d** Detail of **c** showing *T. solium* cells expressing PGRMC exclusively on subtegument cells (arrows) at higher magnification (400×) from shows in. *Abbreviations*: T, tegumental cells; GL, germinal layer. *Scale-bars*: **a**, 10 μm; **b**, 10 μm; **c**, 10 μm; **d**,10 μm; **e**, 10 μm; **f**, 10 μm

Immunofluorescence assays were performed to determinate the presence of the progesterone binding membrane protein in the *T. solium* cysticerci. Cysticerci incubated only in the presence of the isotype antibody and secondary antibody that were used as control, did not give any positive signal related to the progesterone binding membrane protein (Fig. 3a). Our result showed intense fluorescence detected in cysticercus subtegumental cells, revealing that progesterone-binding membrane protein is expressed in parasite cells (Fig. 3b). The distribution of the expression of the positive cells for PGRMC, is surrounding all the tegumental tissue (Fig. 3c). This finding confirmed that experimental conditions were optimal for detecting exclusively tegumental parasite cells expressing progesterone-binding membrane molecules and not false positive signals (Fig. 3d-f).

### 2D-E and localization of PGRMC

**Fig. 4:**
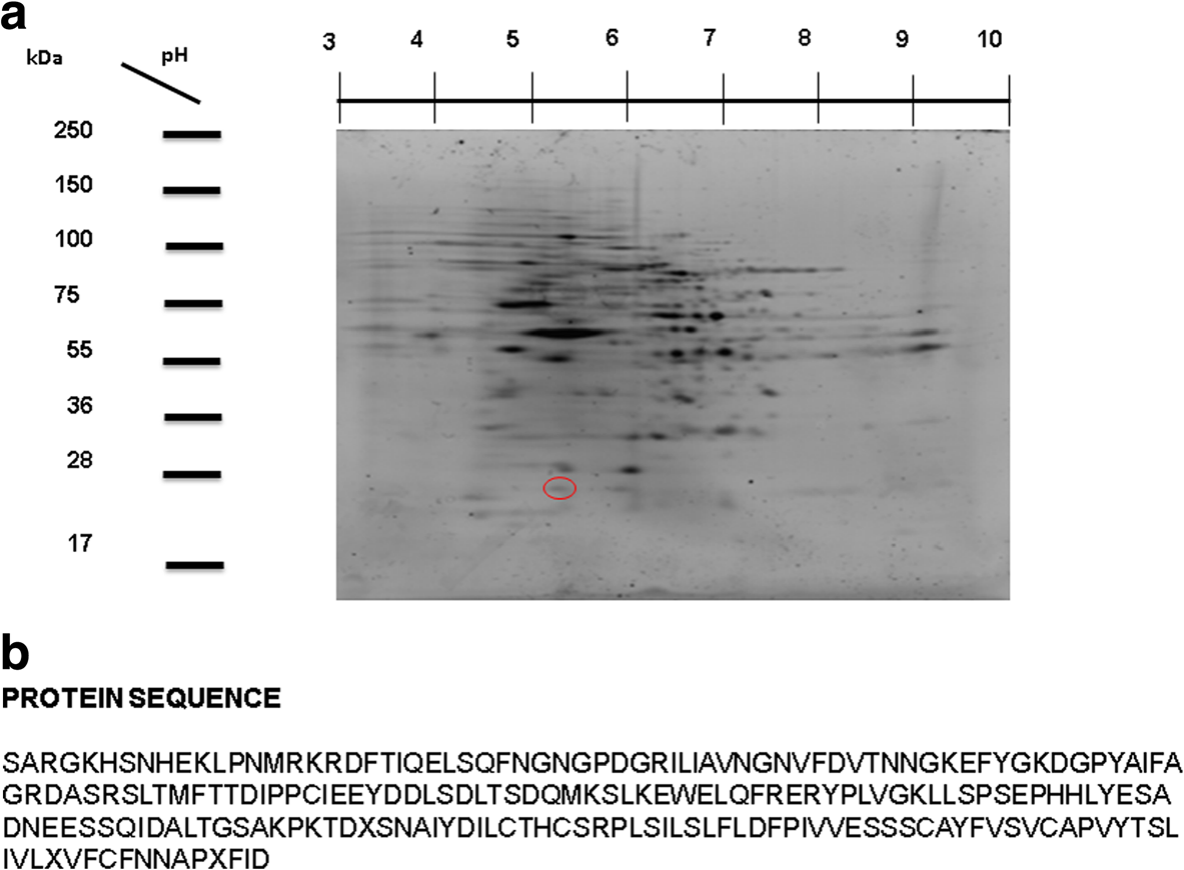
*Taenia solium* PGRMC characterization by 2D–E. **a** A representative 2D-E gel showing the location of the protein spot selected for sequencing (red ellipse). **b** Sequence obtained from protein spot by MALDI-TOF and used in further molecular analysis

Total proteins from *T. solium* cysticerci were separated in a pH range of 3–10, according to their isoelectrical point (IP) and molecular weight (Fig. 4a). Moreover, a well-defined protein spot around pH 5 and 25 KDa was recognized, corresponding to the IP of most of the sequenced PGRMC (Fig. 4a). Sequence of the corresponding protein spot of PGRMC is shown in Fig. 4b. Interestingly, this sequence showed high homology (90%) to those sequences included in the *T. spiralis* genome (GenBank: EFV58821.1) [37]; *S. japonicum* genome (GenBank: CAX73419.1) [38]; *S. haematobium* genome (GenBank: KGB35529.1) [39]; and *E. granulosus* genome (GenBank: CDS20257.1) [40]; and 100% homology to the sequence annotated in the *T. solium* genome (PRJNA170813, Worm Base Parasite) [40].

### Modeling PGRMC and docking to progesterone

**Fig. 5:**
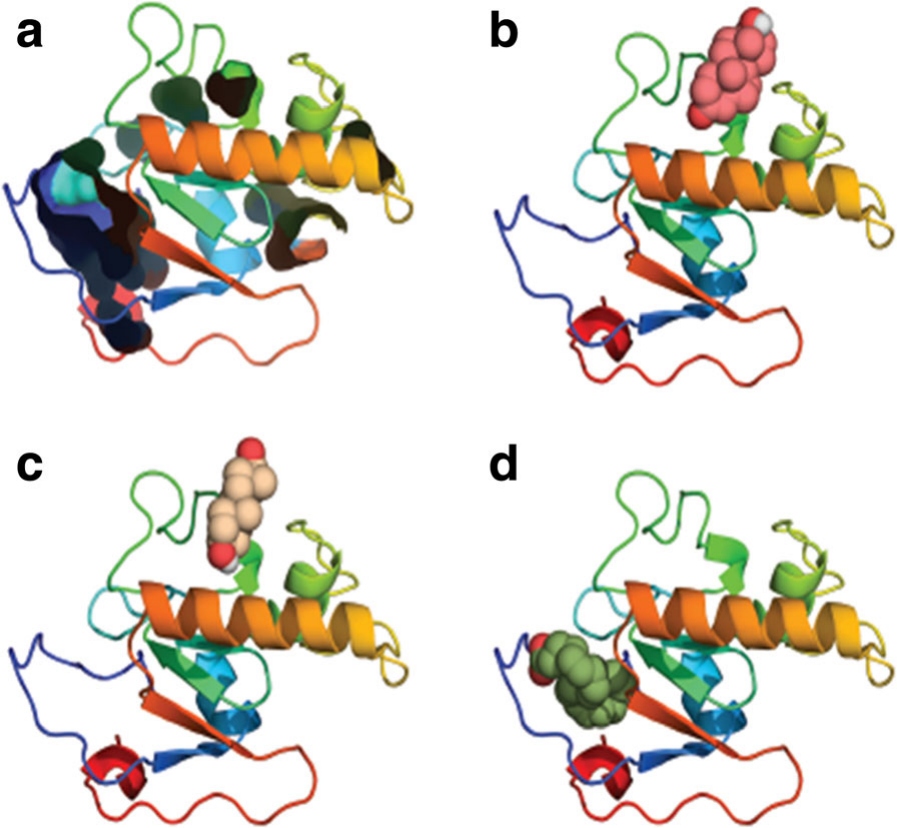
The hydrophilic segment model of PGRMC. The structure is represented as cartoons with N-terminus in blue and the C-terminus in red. In **a** the cavities are represented as surfaces in the progesterone docking, **b** shows docking of testosterone, **c** dehydroepiandrosterone, and **d** arachidonic acid, all of them in a CPK representation. Note that both dehydroepiandrosterone and arachidonic acid bind to a similar site whereas progesterone binds closer to the C-terminus

Homology modeling of the first 136 residues of PGRMC (sequence detailed in Methods) yielded a globular protein containing 19.1% alpha-helix, 14.7% beta-sheet and 25% without any predictive secondary structure (Fig. 5). This domain was found to be similar to a cytochrome b5, member of a family of steroid-binding proteins. Residues 137 to 217 were not modeled since no homology was found to any known protein and were excluded from docking analysis. Results from blind docking are shown in Table 1. Binding of steroids to our model is about 1 kcal/mol higher that to the control molecules, i.e. fatty acids. While steroids consistently found the same binding site (Fig. 5), fatty acids preferentially located either to an unstructured region of the model or to the same binding site as steroids. The binding site identified by docking contains a set of hydrophobic residues: F55, Y62, F77, W105 and T78 and R112 (numbering according to Methods). Since docking allows for the rotation of bonds within the ligand but not for the protein, we performed molecular dynamics simulations starting from the docking results for progesterone and stearic acid (Fig. 5a). The latter was selected as reference for MMGBSA, a method to estimate the relative binding energies (ΔG_bind_). Our simulations show that the binding site located by docking is indeed stable for 100 ns for both ligands, P_4_ and stearic acid. For the actual calculations, only the last 50 ns were used. ΔG_bind_ for P_4_ and stearic acid were estimated as -26.9 ± 2.9 and -25.7 ± 3.8, respectively. Their ΔG_bind_ is -1.2 in line with the -1.4 obtained through docking. It is noteworthy that while their estimated binding energies are similar, the structure of our model experiences a conformational change in the presence of P_4_ but not stearic acid (Fig. 5a), suggesting a conformational change consistent with ligand-induced signaling by P_4_ but not stearic acid (Fig. 5).

**Table 1:** Docking of several hydrophobic compounds to PGRC2. The data presented show the best result for each compound. Score is in kcal/mol, as calculated by Autodock Vina

Rank	Ligand	Score
1	Dehydroepiandrosterone	-7.3
2	Testosterone	-7.2
3	4-Dihydrotestosterone	-7.2
4	Estradiol	-7.1
5	Progesterone	-7
6	β-estradiol	-6.9
7	β-arachidonic	-5
8	Linoleic	-4.9
9	Oleic	-4.7
10	Palmitic	-4.4
11	Stearic	-4.3

### Phylogenetic analysis and sequence alignment

**Fig. 6:**
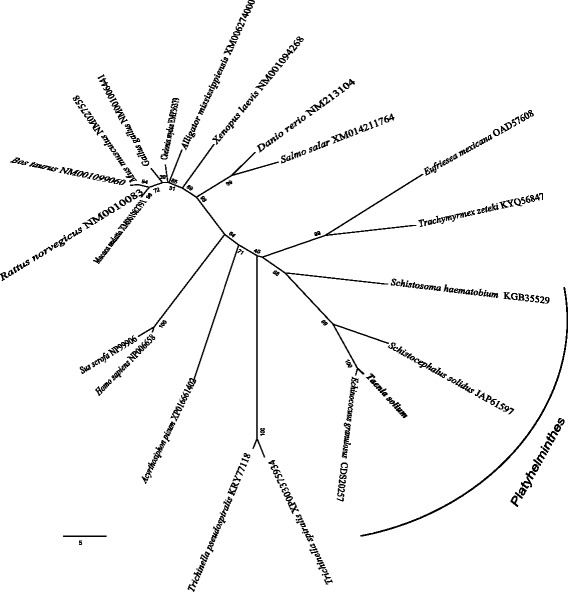
Neighbor-joining tree (NJT) inferred from a dataset composed of 133 characters and with 21 taxa. PGRMCs from several species of fish, amphibians, reptilians, birds and mammals were analyzed through a NJT for searching probable relationship with the *T. solium* PGRMC identified and sequenced. Numbers near internal nodes show bootstrap replicates

The neighbor-joining tree obtained in the present study, brought to light that the progesterone receptor component of *T. solium* is related to a protein present in *E. granulosus* and both are nested within a cluster including other Platyhelminthes such as *Schistocephalus solidus* and *Schistosoma haematobium* with a strong bootstrap support of 85%. The neighbor-joining tree also placed together the two sequences of the progesterone receptor described for nematodes, whereas the three sequences for arthropods and the 12 sequences for vertebrates were spread out in different clusters (Fig. 6). Finally, sequences of the progesterone receptor component in the swine (*Sus scrofa*) and humans conformed a cluster in the tree with 100% bootstrap support. This cluster was very divergent to that progesterone receptor component found in *T. solium* (Fig. 6).

## Discussion

As previously shown, the effects of P_4_ upon scolex evagination and adult worm growth were repeated and confirmed. To this regard, we demonstrated that the possible action mechanism through which P_4_ exerts its actions upon *T. solium* differentiation might involve the binding to a membrane protein as is the case of PGRMC. Our results show the presence of the progesterone binding membrane protein located at the cysticercus cell surface. This result suggests that helminths seem to have developed a molecule able to recognize progesterone (and possible other steroid hormones) with the aim of mediating its hormonal effects.

Interestingly, our results also suggest that the possible mechanism by which P_4_ exerts its actions upon cysticerci differentiation may primarily involve progesterone membrane receptors as well as nuclear PR-like proteins in a second plane. In order to examine the localization of the progesterone binding membrane protein in the *T. solium* cysticerci, immunofluorescence staining was performed. Interestingly, adjacent cells in the cysticerci tegument and subtegument showed intense fluorescent signal suggesting a mechanism where progesterone might be captured from the external environment. The idea is supported by the fact of having found PGRMC expression in cysticerci cells by flow cytometry. We found that *T. solium-*derived cells expressed some protein resembling the PGRMC. This finding may suggest that a putative steroid-binding protein present in the parasite might mediate P_4_ effect in the differentiation process of *T. solium*. Results also showed a slight increase in the amount of PGRMC on cysticercus cells treated with P_4_ as compared to cells derived from parasites cultured under control conditions. This finding suggests that P_4_ is able to increase the PGRMC expression, thus promoting scolex evagination.

Tsai et al. [40] sequenced the *T. solium* genome in 2013 by using the strategy of “shotgun genome sequencing”. From these fragments, construction of genes present in the *T. solium* genome was carried out. These gene constructions opened the possibility of using hardware analysis to detect the presence of different coding sequences contained in the *T. solium* genome. These analyses were performed with bio-computer hardware like EAnnot, SNAP and FgenesH, which in turn are based on algorithms able to find preserved regions including exon-intron junction sites, and polyadenylated sequences. Notably, transcription of both exon-intron junction sites and polyadenylated sequences are partially regulated by the predicted form of the PGRMC [41]. As mentioned in the result section, this sequence 5′ and 3′ seems to have lost the non-coding regions (UTR’s) at the end as well polyadenylation sites [41].

In silico analysis regarding the predicted protein sequence of this transcript, gives place to an open reading frame corresponding to the whole sequence of RNA. The corresponding amino acid sequence was accurately translated using this sequence, by carrying out in silico analysis on the protein topology. Also, 2D–E coupled to mass-spectroscopy sequencing revealed the PGRMC sequence that was used to estimate its binding ability and possible evolutionary origin. As shown in Fig. 5, PGRMC has two domains corresponding to extra and intracellular compartments. This is consistent with previous works reporting PGRMC-1 and PGRMC-2 found in other species [41–43].

In parallel, using the BLAST platform to find conserve domains in this protein sequence, we determined that PGRMC has a binding domain to steroids in the C-terminal tail (118-to-168 aa sequence). To some extent, present finding disagrees with a previous study where authors claimed the binding domain to steroids is a transmembrane domain [44]. Interestingly, the free-living nematode *C. elegans* presents DAF, which have a nuclear location, and different alleles of the gene that codifies for the receptor, and express those genes depending of the larvae stage of the nematode [20].

Furthermore, we determined that molecular weight of PGRMC in *T. solium* is 24.2 kDa, with isoelectric point around 4.9. In general, the known progesterone receptor membrane components range from 18 to 25 kDa. The *T. solium* PGRMC was detected in the parasite tegument that is the tissue in close contact to the host microenvironment. PGRMC-2 has been mainly detected on reproductive tissues of mammals, including ovary, endometrium, and placenta, where it has been associated with multiple cellular events such as maturation, differentiation and proliferation [42].

Escobedo et al. [15] demonstrated that progesterone, estradiol and testosterone have a direct effect on the *T. solium* cysticercus evagination. Specifically, progesterone increases evagination and parasite growth, whereas androgens induces parasite death [45]. Such different actions might be partially explained due to the fact that PGRMC appears to bind all these steroids with different affinity, which in turn is able to modify its cellular functions, as can be seen in Table 1.

The neighbor-joining tree supported that PGRMC of *T. solium* is closely related to that of *E. granulosus*; both in turn are nested within a cluster, comprising other plathelminths such as *S. solidus* and *S. haematobium*. On the other hand, PGRMC sequences of the natural hosts (pigs and humans) were nested in a different cluster showing significant divergence with respect to *T. solium*.

## Conclusions

A possibly functional PGRCM was found in *T. solium* and is described here. Whether the gene was acquired by *T. solium* through horizontal gene transfer or evolved by mimicry, or simply from common ancestral genes, remains to be elucidated. Our findings provide evidence on the crosstalk between host and parasite at molecular and evolutionary levels, providing new information which may be useful in designing anti-helminthic drugs, with the aim of specifically recognizing parasite cells with minimal secondary effects to the host.
